# On the sensitivity of patient‐specific IMRT QA to MLC positioning errors

**DOI:** 10.1120/jacmp.v10i1.2915

**Published:** 2009-02-05

**Authors:** Guanghua Yan, Chihray Liu, Thomas A Simon, Lee‐Cheng Peng, Christopher Fox, Jonathan G Li

**Affiliations:** ^1^ Department of Radiation Oncology University of Florida Gainesville FL U.S.A.; ^2^ Department of Nuclear and Radiological Engineering University of Florida Gainesville FL U.S.A.

**Keywords:** IMRT, dosimetry, quality assurance, MLC positioning error

## Abstract

Accurate multileaf collimator (MLC) leaf positioning plays an essential role in the effective implementation of intensity modulated radiation therapy (IMRT). This work evaluates the sensitivity of current patient‐specific IMRT quality assurance (QA) procedures to minor MLC leaf positioning errors. Random errors of up to 2 mm and systematic errors of ±1mm and ±2mm in MLC leaf positions were introduced into 8 clinical IMRT patient plans (totaling 53 fields). Planar dose distributions calculated with modified plans were compared to dose distributions measured with both radiochromic films and a diode matrix. The agreement between calculation and measurement was evaluated using both absolute distance‐to‐agreement (DTA) analysis and γ index with 2%/2mm and 3%/3mm criteria. It was found that both the radiochromic film and the diode matrix could only detect systematic errors on the order of 2 mm or above. The diode array had larger sensitivity than film due to its excellent detector response (such as small variation, linear response, etc.). No difference was found between DTA analysis and γ index in terms of the sensitivity to MLC positioning errors. Higher sensitivity was observed with 2%/2mm than with 3%/3mm in general. When using the diode array and 2%/2mm criterion, the IMRT QA procedure showed strongest sensitivity to MLC position errors and, at the same time, achieved clinically acceptable passing rates. More accurate dose calculation and measurement would further enhance the sensitivity of patient‐specific IMRT QA to MLC positioning errors. However, considering the significant dosimetric effect such MLC errors could cause, patient‐specific IMRT QA should be combined with a periodic MLC QA program in order to guarantee the accuracy of IMRT delivery.

PACS numbers: 87.50.Gi, 87.52.Df, 87.52.Px, 87.53.Dq, 87.53.Tf, 87.53.Kn, 87.56.Fc

## I. INTRODUCTION

Intensity modulated radiation therapy (IMRT) has become the treatment technique of choice for many types of cancers receiving radiation therapy. The clinical efficacy of IMRT relies on dose escalation to the tumor while avoiding toxicity to the surrounding critical structures. Accurate multileaf collimator (MLC) leaf positioning plays a crucial role in the effective implementation of MLC‐based IMRT. Tolerance limits for leaf position accuracy and reproducibility have been suggested for IMRT^(1)^ which are more stringent than for conventional radiation therapy.^(2)^

Several authors have studied the dosimetric effect of leaf positioning errors.^(^ ^3^ ^–^ ^7^ ^)^ Luo et al.^(3)^ studied the correlation between leaf position errors and dosimetric impact in prostate cancer treatment. They found a linear correlation between the target dose error and the average MLC position error, with 1% target dose change arising from 0.2 mm systematic leaf position errors. LoSasso et al.^(4)^ also reported that a 0.2 mm gap variation leads to 1% dose variation with an average gap width of 2 cm with dynamic beam delivery.^(8)^ Mu et al.^(5)^ studied the dosimetric effect of leaf position errors on head and neck patients by deliberately introducing random (uniformly sampled from 0 mm, ±1mm and ±2mm) and systematic (±0.5mm or ±1mm) leaf positioning errors into the plan. They found no significant dosimetric effect (<2% dose change to both target and critical organs) introduced by random leaf position errors up to 2 mm, while significant effects (8% change in $D_{95\%}$ and approximately 12% in $D_{0.1\text{cc}}$ to critical organs) were observed by 1 mm systematic leaf position errors in complex IMRT plans. Zygmanski et al.^(6)^ studied the dosimetric effect of truncated Gaussian (with 0.1 cm standard deviation) shaped random leaf position errors. They found that although the average composite dose to the target of a nine field IMRT plan was changed only by 3%, fluence change resulting from each single field was commonly >10%. Woo et al.^(7)^ found that leaf position uncertainty could lead to dose variations of up to 13% when positioning the ion chamber on the field edge. All these studies emphasized the importance of the MLC positioning accuracy and reproducibility.

Several authors have reported excellent accuracy of MLC leaf position by analyzing MLC log files for both dynamic MLC and static MLC.^(3)^ ^,^ ^(6)^ ^,^ ^(8)^ For dynamic MLC, Zygmanski et al.^(6)^ reported <0.05cm leaf position error, while LoSasso et al.^(8)^ found that the average leaf gap error was much smaller than 0.02 cm. For static MLC, Luo et al.^(3)^ reported average leaf position errors of approximately 0.05 cm based on the analysis of MLC log files. On the other hand, by using a fast video‐based electronic portal imaging device, Zeidan et al.^(9)^ observed a maximum unplanned leaf movement of 3 mm during static MLC delivery. Another study by the same group, based on MLC log file analysis, reported that in approximately 80% of the total segment deliveries, at least one collimator leaf had unplanned movement of at least 1 mm (projected at isocenter) during segment delivery.^(10)^ Significant dosimetric impact could arise from these MLC position errors which suggests that periodic MLC quality assurance (QA) should be performed to ensure the accuracy and reproducibility of MLC leaf positions.

It is both challenging and time‐consuming to check the position accuracy of every single MLC leaf pair at all possible off‐axis positions.^(^ ^11^ ^–^ ^14^ ^)^ In practice, dedicated MLC QA is conducted bi‐weekly, or even less frequently, whereas patient‐specific IMRT QA is usually done for every new patient before the start of IMRT treatment. The aim of this work is to assess the sensitivity of patient‐specific IMRT QA to leaf position errors. A common method of patient‐specific QA is to recalculate the treatment plan in a QA phantom with all beams at 0° gantry angle (IEC convention) and normal to the phantom surface. The planar dose distribution is measured under the same geometry using film or two‐dimensional (2D) detector arrays. The agreement between the calculated and measured planar dose distributions is then quantified using parameters like the Gamma index,^(15)^ percent dose difference (%Diff), or distance‐to‐agreement (DTA).

Patient‐specific IMRT QA is required for every new IMRT patient to ensure the accuracy of the treatment plan. Childress et al.^(16)^ have investigated the feasibility of using a 2D dosimetric system to detect gross errors such as beam energy change, wrong patient's beam data, one beam collimator angle change of 90°, gantry angle change of 10°, and omitting the delivery of one beam. However, the sensitivity of commonly used patient‐specific IMRT QA systems to subtle MLC position errors has not been fully examined. Sastre‐Padro et al.^(17)^ studied the consequences of subtle leaf positioning errors on IMRT delivery, but whether the errors could be detected by patient‐specific IMRT QA systems was not answered.

In this work, random MLC position errors up to 2 mm as well as systematic errors (±1mm, ±2mm) were introduced into treatment plans for 8 H&N IMRT patients (totaling 53 fields).

Planar dose distributions calculated before and after introducing errors were compared to dose distributions measured with both film and 2D diode arrays using the gamma index as well as DTA criterion on a field‐by‐field basis. The change in the passing rate distribution was used as an indicator to study the sensitivity of the IMRT QA procedure to MLC position errors.

## II. MATERIALS AND METHODS

### A. Patient plan selection

Eight head and neck IMRT plans (total of 53 fields) previously used for patient treatment with 6 MV photon beams of a linear accelerator (Trilogy, Varian Medical Systems, Palo Alto, CA) with 120‐leaf Millennium MLC were randomly selected. The step‐and‐shoot treatment plans were generated with a commercial treatment planning system (TPS, Pinnacle^3^, version 8.0d, Philips Medical Systems, Madison, WI) with direct machine parameter optimization (DMPO) option that directly optimizes the shape and weight of each MLC segment. Minimum segment area and minimum segment MU were set to 4 cm^2^ and 3 MU, respectively. The adaptive convolution dose calculation algorithm with inhomogeneity correction was used for all the plans.

### B. Leaf positioning errors simulation

Random and systematic leaf positioning errors were simulated in the same manner as in Mu et al.^(5)^ The original treatment plans were exported from Pinnacle using the Pinnacle scripting language. Leaf position errors were introduced into the plans by directly modifying the leaf positions in the exported treatment plans. Random errors were uniformly sampled from [0 mm, ±1mm, ±2mm] for each leaf from both leaf banks. Negative and positive errors make the leaf end‐to‐end distance smaller and larger, respectively. When leaf collisions happen, the leaves were assume closed and they were assigned the same position. When introducing systematic errors, the position of each open leaf was changed by the same amount (±1mm and ±2mm). A total of 6 plans were generated for each patient: the original plan without leaf positioning error, the plan with random errors, and four plans with different amount of systematic errors. The modified treatment plans were imported back into Pinnacle for planar dose calculation. Planar dose distribution was calculated for each field at 10 cm depth and 90 cm source‐to‐surface distance (SSD) in a rectangular water phantom with 0° gantry angle (IEC convention) and normal to the phantom surface. All the calculation was done using a $1.0\times 1.0\;{\text{mm}}^{2}$ dose grid.

### C. Dose distribution measurement

In standard practice for patient‐specific IMRT QA, both film and 2D detector arrays are widely used. In this work, both radiochromic films and a 2D diode array were employed to measure the planar dose distribution under the same geometry as in calculation. Radiochromic films (Gafchromic EBT film, ISP Corp., Wayne, NJ) were sandwiched between solid water pieces at 10 cm depth with a source‐to‐film distance of 100 cm. Small marks were placed on the edges of the films for geometric registration. After irradiation, the films were left for 24 hours before analysis (as recommended by Niroomand‐Rad et al.^(18)^) to minimize post irradiation coloration effects. A commercial flatbed scanner (EPSON 1680) was used to digitize the film at a resolution of 100 μm/pixel and was sampled at a spatial resolution of 1 mm at both directions using commercial data analysis software (Matlab v.7.0, Mathworks, Inc.). An adaptive 2D wiener filter of 4×4 pixel region was used to reduce the inherent noise. Film images were acquired from the red CCD channel only. Sensitometric curves were obtained by delivering varying amounts of radiation which covered the range of the doses of interest. When irradiating the radiochromic films, all doses were rescaled such that the maximum dose was around 100 cGy to avoid the uncertainty at low doses.^(19)^

Convenient 2D detector arrays are replacing film for patient‐specific IMRT QA. A 2D diode matrix (MapCHECK 1175, Sun Nuclear Corp., Melbourne, FL) was also used to measure the planar dose distribution under the same geometry as the film measurement. The MapCHECK device has been demonstrated to be an excellent tool for the verification of IMRT fields with little energy or dose rate dependence.^(^ ^20^ ^–^ ^21^ ^)^ An intercomparison between film and MapCHECK showed that MapCHECK can effectively replace film dosimetry in routine IMRT QA, even though it has a limited spatial resolution.^(22)^ The MapCHECK device was calibrated for absolute dose measurement.

The original clinical treatment plans without MLC positioning errors were delivered for measurement. Each of the 53 IMRT fields was measured with the diode array and 10 of the fields were measured with the radiochromic films.

### D. Planar dose comparison

Planar dose distributions with and without the leaf position errors were calculated for each of the 53 fields. The calculated planar dose distributions were imported into the MapCHECK analysis software^(20)^ and compared with either MapCHECK measurement or film measurement. When calculation was compared with film measurement, film measurement was loaded into MapCHECK software and used as reference. Each of the MapCHECK (or film) measured points above 10% of the maximum dose level was compared with calculation using absolute distance‐to‐agreement (DTA) comparison as well as γ index in the absolute dose comparison mode.^(15)^ ^,^ ^(23)^ Two sets of criteria, 2%/2mm and 3%/3mm, were employed, and the percentage of the points passing the acceptance criteria was evaluated.

### E. Criteria for identifying errors

The decision regarding whether the procedure could identify the MLC errors was made based on the comparison between passing rate distributions before and after the errors were introduced. In this work, the change in average passing rates and a significance test were used to make the decision. A sudden drop of the average passing rate from the baseline value serves as a first indication of errors in the system. The threshold of average passing rate decrease was arbitrarily set at 5%. A non‐parametric significance test, Wilcoxon rank‐sum test,^(24)^ was employed to compare the distribution of the passing rates before and after the introduction of the MLC position errors. The Wilcoxon rank‐sum test was chosen over the popular student t‐test because the passing rates didn't follow the normal distribution and the standard deviation varied significantly. The p‐value of the test gives the probability that the two compared variables are from the same distribution with equal median. The significance level of the test was set to 0.01. In other words, the difference was significant only when the p‐value of the test was less than 0.01. In summary, the introduced MLC position errors would be considered “identified” when (1) the drop in average passing rates was larger than 5%, and (2) the p‐value from the Wilcoxon rank‐sum test was smaller than 0.01.

## III. RESULTS

**Figure 1:**
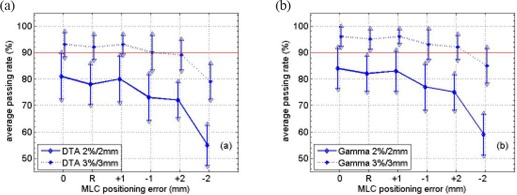
Average passing rates when comparing Pinnacle calculated planar dose distribution and Gafchromic Ebt film measurement using 2%/2mm and 3%/3mm criterion with (a) Dta analysis and (b) the γ index for a total of 10 Imrt fields. The x‐axis, from left to right, stands for no MLC positioning error, random errors and systematic errors of ±1mm and ±2mm, respectively. The error bar indicates one standard deviation.

**Figure 2:**
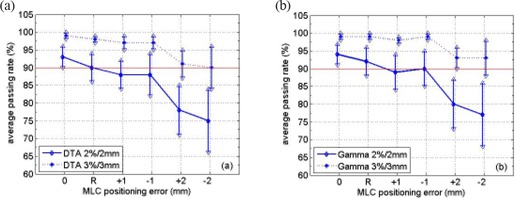
Average passing rates when comparing Pinnacle calculated planar dose distribution and MapCHECK measurement using 2%/2mm and 3%/3mm criterion with (a) DTA analysis and (b) the γ index for a total of 53 IMRT fields. The x‐axis, from left to right, stands for no MLC positioning error, random errors and systematic errors of ±1mm and ±2mm, respectively. The error bar indicates one standard deviation.

**Figure 3:**
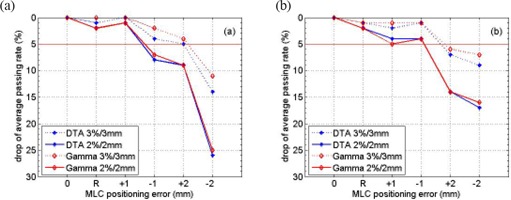
Drop of average passing rates with respect to the clinical plan (no MLC positioning errors) with the introduction of random and systematic errors. The comparison was between Pinnacle calculated planar dose distribution and (a) film or (b) MapCHECK measurement using DTA 2%/2mm and 3%/3mm as well as γ 2%/2mm and 3%/3mm criterion. The x‐axis, from left to right, stands for no MLC positioning error, random errors and systematic errors of ±1mm and ±2mm, respectively.

The average passing rates and the standard deviations using both films and the diode array are displayed in Figs. 1 and 2, respectively, for different MLC positioning errors. The drop of average passing rate caused by MLC positioning errors is shown in Fig. 3. The p‐value from the Wilcoxon rank‐sum test is summarized in Table 1. For film measurements, the average passing rate with “DTA, 3%/3mm” criterion was 93%±5% (1 standard deviation) without MLC errors. With random leaf position errors of up to 2 mm and systematic errors on the order of 1 mm, the average passing rate dropped by less than 5% and was still around 90%. For 2 mm systematic errors, an asymmetric behavior was observed with a 5% drop in average passing rate for positive errors but an approximately 15% drop for negative errors. The p‐values from the rank‐sum test indicated significant difference only for negative 2 mm systematic error, with a p‐value of 1.10E–03. In this case, we conclude that only the −2mm systematic MLC positioning error could be identified by patient‐specific IMRT QA procedure when using EBT film and “DTA, 3%/3mm”.

**Table 1:** P‐values from two‐tailed Wilcoxon rank‐sum test. The test compared the distribution of passing rates before and after the introduction of Mlc position errors.

			*Random*	*1 mm*	*p‐Value* −1mm	*2 mm*	*‐2 mm*
EBT Film	DTA	2%/2mm	3.28E‐01	9.59E‐01	1.05E‐01	2.81E‐02	6.22E‐04
		3%/3mm	5.56E‐01	7.98E‐01	1.61E‐01	7.74E‐02	1.10E‐03
	Gamma	2%/2mm	5.05E‐01	9.81E‐01	1.30E‐01	2.81E‐02	6.22E‐04
		3%/3mm	6.29E‐01	1.00E+00	3.14E‐01	9.95E‐02	1.90E‐03
MapCHECK	DTA	2%/2mm	1.10E‐02	1.29E‐05	2.60E‐03	2.35E‐11	6.86E‐10
		3%/3mm	3.20E‐02	1.90E‐05	3.60E‐03	2.84E‐11	1.62E‐08
	Gamma	2%/2mm	1.76E‐02	1.08E‐05	3.45E‐04	3.97E‐11	3.28E‐10
		3%/3mm	7.59E‐05	3.85E‐06	3.57E‐04	4.33E‐12	9.20E‐10

When using “DTA, 2%/2mm” criterion in film result analysis, both the −1mm and ±2mm systematic errors would cause the average passing rates to drop by more than 5%. But rank‐sum test showed that only the −2mm error caused significant difference in passing rate distribution with a p‐value of 6.22E–04. For the other cases, there is significant overlap between the distributions. Based on our criteria for identifying errors, only the −2mm errors could be identified. The same can be concluded when employing gamma test for film result analysis.

Fig. 2 shows the results when using MapCHECK as the detector. The drop of average passing rates is displayed in Fig. 3(b) and the p‐values from Wilcoxon rank‐sum test are summarized in Table 1. The p‐values indicated that both ±1mm errors and ±2mm errors caused significant difference in the passing rate distributions for both 2%/2mm and 3%/3mm criterion, using either DTA comparison or γ analysis. However, Fig. 3(b) shows that larger than 5% drop in average passing rate was observed only for ±2mm error. Thus we conclude that with MapCHECK, the IMRT QA procedure could identify systematic errors on the order of 2 mm.

## IV. DISCUSSION

Due to the complexity of planning and delivery of IMRT, patient‐specific QA is recommended for every new patient before the start of IMRT delivery. This could potentially detect any gross errors such as the wrong beam energy, wrong patient plan, or any data transfer errors from the TPS to the delivery system. However, the ability of patient‐specific QA in detecting subtle errors, such as TPS calculation error or MLC positioning error, is more difficult to quantify. In practice, after commissioning of the IMRT planning and delivery system, baseline values for patient‐specific QA acceptance criteria, which might be site‐dependent, could be established over a period of time. A sudden drop in passing rates would signify deviations in one or more of the IMRT planning and delivery components and would warrant further investigation. Therefore, whether the commonly used QA method of field‐by‐field planar dose comparison is sensitive to MLC positioning errors on the order that would significantly affect the treatment is of practical interest.

Both radiochromic film and the MapCHECK device were used in this study. When no MLC positioning errors were introduced, the passing rate distribution from the MapCHECK device had less variation than radiochromic film: 1% vs. 5% with the 3%/3mm criterion, and 3% vs. 9% with the 2%/2mm criterion. With larger MLC positioning errors, the variation associated with MapCHECK passing rate distribution gradually increased to around 6% with 3%/3mm and 9% with 2%/2mm, while it stayed constant for radiochromic film. If the average passing rates didn't decrease significantly when the MLC errors were introduced, the passing rate distribution would overlap the one without errors. Figs. 1 and 2 show that the overlapping was larger for film than for the MapCHECK device due to the larger variation associated with film. This fact should explain the larger p‐value from rank‐sum test with radiochromic film than with MapCHECK. From this perspective, the MapCHECK device showed larger sensitivity to MLC positioning errors than radiochromic film. The difference in variation associated with passing rate distribution was mainly due to different detector response. The variation of film sensitivity is on the order of 1.5% to approximately 4% depending on dose level,^(19)^ while MapCHECK has a variation of only 0.15%.^(^ ^20^ ^–^ ^21^ ^)^

Larger variation in the passing rate distribution was found when using 2%/2mm than 3%/3mm. At the same time, a larger drop in the average passing rate was noticed with 2%/2mm than with 3%/3mm as shown in Fig. 3. Table 1 shows smaller p‐values with 2%/2mm than with 3%/3mm in general, which meant a higher probability of distinguishing the passing rate distributions with 2%/2mm. Therefore, a tighter criterion shows more sensitivity to MLC position errors. With 2%/2mm, the average passing rate was approximately 93% with MapCHECK and approximately 80% with radiochromic film. It was relatively difficult to achieve acceptable passing rates using film with tighter criterion.^(19)^

Both the DTA analysis and γ index were studied in this work. DTA analysis is more stringent than the γ index and on average gives 2% to approximately 3% lower passing rates. However, no significant difference was found in the decrease of average passing rate and the p‐values from rank‐sum test were in general on the same order, which suggested that the choice of using DTA analysis or the γ index didn't affect the sensitivity of the IMRT QA to MLC position errors.

Asymmetric response to +2mm and −2mm systematic MLC positioning errors was noticed with both MapCHECK and film. The decrease in average passing rate was approximately 3% larger with −2mm systematic errors than with +2mm errors when using MapCHECK as the detector. When using film, a 10% larger decrease in average passing rate was noticed with −2mm errors than with +2mm errors (25% vs. 10% when using 2%/2mm criterion for both DTA analysis and the γ index). To investigate the asymmetric behavior with respect to systematic MLC positioning errors, the following simulation was conducted to reveal the cause of such behavior.

**Figure 4:**
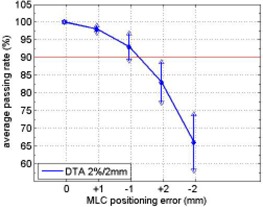
Average passing rates when comparing error free plans (re‐sampled according to MapCHECK detector positions) to plans with +/−1mm and +/−2mm systematic MLC errors using DTA 2%/2mm criterion. The error bar stands for one standard deviation.

The planar dose distribution calculated from plan without MLC errors was re‐sampled and only those points corresponding to a detector position on the MapCHECK device were kept. The re‐sampled dose distribution was compared to other four dose distributions from plans with systematic MLC errors (±1mm and ±2mm) using 2%/2mm DTA criterion. The average passing rates of 15 IMRT plans were shown in Fig. 4. An obvious asymmetric behavior was noticed. Compared to an error free plan, plans with −1mm systematic MLC errors had approximately 3% lower average passing rates than plans with +1mm systematic MLC errors (93% vs. 96%). For 2 mm systematic MLC errors, 17% lower average passing rates were observed for plans with negative errors than plans with positive errors (66% vs. 83%). A similar trend was observed when comparing error‐free plans to plans with errors in full resolution (i.e., no re‐sampling). This simulation exercise showed that the asymmetric behavior is not caused by MLC calibration error or TPS modeling errors. Rather it indicated that the dose distribution is more sensitive to negative systematic MLC errors (which makes the MLC segment opening smaller) than positive errors. The magnitudes of the passing rate differences between the negative and positive MLC errors are very similar to those observed in Figs. 1 and 2.

Childress et al.^(16)^ investigated the feasibility of automatically detecting both gross delivery errors and small delivery errors (5 mm lateral shift of isocenter) with patient‐specific IMRT QA procedures. They found that most scalar dose comparison metrics, including the γ index, failed in identifying isocenter shift error of 5 mm. Our results indicated that the studied IMRT QA procedures could detect systematic MLC positioning errors of 2 mm. A major difference between the Childress study and ours was that the former compared composite dose distribution while our analysis was done on a field‐by‐field basis. One reason for poor detection ability when using composite dose distribution in comparison could be attributed to the fact that when composite dose distribution was analyzed, errors from individual fields could compensate each other.

As TPS beam modeling, dose calculation and dose measurement become more accurate, tighter criterion could be employed in the analysis and still achieve good agreement between calculated and measured dose distribution,^(25)^ which will lead to better and earlier IMRT delivery error detection, especially with MapCHECK since it provides enhanced sensitivity compared with EBT film. However, considering the dosimetric impact of such errors, patient‐specific IMRT QA should be combined with a periodic MLC positioning check program^12^ ^–^ ^14^ in order to guarantee the accuracy of IMRT delivery.

## V. CONCLUSIONS

In this work, we investigated the sensitivity of patient‐specific IMRT QA procedure to MLC positioning errors. Random errors of up to 2 mm and systematic errors of ±1mm and ±2mm were simulated in the treatment plans. Planar dose distributions calculated with plans containing errors were compared to dose measurement with both Gafchromic EBT films and the MapCHECK device, acquired while irradiating the original error‐free plan. DTA analysis and the γ index with 2%/2mm or 3%/3mm criteria were employed to evaluate the agreement between calculation and measurement. The change of passing rates distribution was used as an indicator of the sensitivity to simulated MLC positioning errors. It was found that the studied patient‐specific IMRT QA procedure with both radiochromic film and the MapCHECK device was only able to detect systematic errors on the order of 2 mm. Significantly larger sensitivity to MLC position errors was found with the 2%/2mm criterion than with the 3%/3mm criterion. Since clinical acceptable agreement between calculation and measurement using film dosimetry with 2%/2mm is relatively difficult to achieve, MapCHECK is more appropriate to be employed with a tight criterion. As the accuracy of dose calculation and measurement is further improved, the sensitivity of IMRT QA procedure to MLC positioning errors will be greatly enhanced. However, considering the dosimetric impact of such errors, patient‐specific IMRT QA should be combined with a periodic MLC QA program in order to guarantee the accuracy of IMRT delivery.
